# The Impact of Education Level on Individual Lifestyle Behaviors among Dietetics Students and Professionals

**DOI:** 10.3390/clockssleep6010007

**Published:** 2024-02-10

**Authors:** Joanna Popiolek-Kalisz, Cansu Cakici, Karolina Szczygiel, Agata Przytula

**Affiliations:** 1 Clinical Dietetics Unit, Department of Bioanalytics, Medical University of Lublin, ul. Chodzki 7, 20-093 Lublin, Poland; 2 Department of Cardiology, Cardinal Wyszynski Hospital in Lublin, al. Krasnicka 100, 20-718 Lublin, Poland; 3 Department of Nutrition and Dietetics, Institute of Health Sciences, Aydın Adnan Menderes University, 09010 Efeler, Aydın, Turkey; dytcansucakici@gmail.com

**Keywords:** nutritional education, sleep behavior, nutritional behavior, dietetics

## Abstract

Lifestyle and habits are acquired in the family environment and then shaped by the potential influence of the environment and received education. In recent years, there has been growing interest in understanding the relationship between sleep and dietary behaviors in various health professionals, including medical and dietetics professionals and students, as well as their self-perceived knowledge and attitudes. Despite the importance of this topic, there is a lack of research on the assessment of individual behaviors in dietetics students and professionals. The aim of this study was to assess the impact of education level on individual behaviors regarding nutrition, sleep, and physical activity in dietetics students and professionals. 71 dietetics students and professionals were enrolled in this study. Their overall knowledge, sleep, and nutritional behavior were assessed with a validated Questionnaire of Eating Behaviors at the beginning of their dietetics university education and then prospectively after a year. It was also compared to dieticians who already graduated. The analysis showed that the educational level did not correlate with sleep length or the physical activity level. However, the educational level was correlated with dietary knowledge and properly self-assessed by the participants. Significant differences were observed in both the prospective and comparative analyses. The educational level and knowledge were not correlated with eating behaviors. The self-assessment of nutritional behaviors also did not correlate with the objective assessment. Sleep length did not correlate with BMI, but it was inversely correlated with overall and healthy diet scores and knowledge levels. On the other hand, physical activity levels were positively correlated with healthy diet scores. Dietary education results in better nutritional knowledge; however, it does not significantly impact individual nutritional behaviors among dietetics students and professionals. Moreover, the inverse relationship between sleep length and nutritional knowledge and behaviors, as well as the positive relationship between physical activity level and dietary behaviors, shows that nutritional aspects of lifestyle are probably prioritized among dietetic students and professionals, with an acknowledgment of the role of physical activity and a neglect of sleep hygiene importance. Dietetics students should be advised to use their theoretical knowledge not only to guide their patients but also to implement it in their own lives.

## 1. Introduction

Many factors such as genetic characteristics, age, gender, education level, physical activity level, social and environmental conditions, stress, and working conditions affect an individual’s nutritional status [1]. Individual lifestyle habits, including sleep hygiene and nutrition, are acquired in the family environment and then shaped by the influence of the education received and the environment [2]. In order to acquire healthy habits, it is potentially important that a person has sufficient nutritional knowledge [3]. It is possible to have nutrition knowledge with proper nutrition education [4,5,6]. Due to moving away from their family home and staying in dorms, apartments, etc., university students experience some changes in their everyday habits. The habit of cooking meals at home and consuming home-cooked food is replaced by more practical, easily accessible, and unhealthy fast food as students’ time at home decreases [7,8]. Moreover, the COVID-19 pandemic could impact behaviors regarding sleep hygiene in young adults, which can result from psychological changes such as higher depression and anxiety rates, and even pro-inflammatory immune responses [9,10,11]. On the other hand, there is evidence that sleep hygiene knowledge can positively impact sleep quality [5].

Dietetics professionals and students play a crucial role in promoting healthy lifestyle habits and preventing chronic diseases. However, even though sleep quality is a factor related to mortality and the incidence of selected diseases, it is often neglected among other lifestyle components [12,13,14]. In recent years, there has been growing interest in understanding individual behaviors of various health professionals, including medical and dietetics professionals and students, as well as their self-perceived knowledge and attitudes [6]. Several studies have explored the behaviors of medical students and physicians, revealing gaps in their knowledge and attitudes, which may impact their ability to provide lifestyle counseling to patients [15,16,17]. It is crucial for people working in the medical and nutrition fields to evaluate their own individual knowledge and behaviors. Self-assessment tools can provide valuable insights into areas where individuals may need to improve their own lifestyle habits or knowledge to better support their patients and clients. Similarly, studies have also examined the nutritional behaviors and knowledge of dietetics professionals and students, highlighting the need for continued education and training in this field [17,18,19,20,21]. Furthermore, the self-assessment of nutritional behaviors and knowledge has been shown to be an effective tool for promoting behavioral change and improving overall dietary quality [22,23]. However, according to studies, dietitians may not always put their advice into practice. It is important to note that nutritional behavior is not the only factor that can impact obesity incidence, as it was shown that short sleep is a risk factor for obesity in children and adults [14]. Moreover, studies have found that dietetics students and professionals have similar dietary habits to the general population, with inadequate intakes of fruits, vegetables, and whole grains and an excessive intake of saturated fat, added sugars, and sodium [24,25]. As a result of their accessibility and affordability, they also tend to consume fried foods, sweets, fizzy drinks, and fast food, according to another study [26].

Despite the importance of this topic, there is a lack of research on the assessment of individual behaviors in dietetics students and professionals throughout their education path. Therefore, the present study aims to address this gap in the literature by prospectively examining the sleep and dietary behaviors and self-perceived nutritional knowledge and attitudes of dietetics students and professionals. Our hypothesis was that academic education in the nutritional field does not impact individual lifestyle behaviors (dietary, sleep, and physical activity). In addition, the implications of the findings of this study for clinical practice are discussed, and the importance of continuing education and focusing on multiple aspects of civilizational diseases, including sleep hygiene, for healthcare professionals is emphasized.

## 2. Results

### 2.1. Characteristics of the Study Group

The group included 71 dietetics students (37 bachelor’s and 34 master’s course students). In the bachelor’s course, men constituted 21.6%, while in the master’s course, men constituted 14.7%, with 18.3% in the overall group, which is probably caused by the greater interest in nutrition expressed by women compared to men. The mean age of the participants was 20.7 ± 2.8 years. The mean body mass was 63.74 ± 13.6 kg, the height was 169.79 ± 9.03 cm, and the BMI was 21.69 ± 3.75 kg/m^2^. During weekdays, 15.9% of the participants sleep less than 6 h, 79.5% sleep 6–9 h, and 4.5% sleep more than 9 h. During weekends, 5.7% sleep less than 6 h, 60.2% sleep between 6 and 9 h, and 34.1% sleep more than 9 h. Regarding individual nutritional behaviors, the mean DQI was 20.57 ± 12.09 and the mean nutritional knowledge test result was 17.71 ± 3.33 points.

### 2.2. General Nutritional Knowledge and Eating Behavior

The prospective comparison among the bachelor’s student group showed that there were no significant differences in their nutritional knowledge level, BMI, Diet Quality Index (DQI), and Non-Healthy Diet Index (nHDI); however, there was a significant difference after a year of education, as the students were characterized by a lower Pro-Healthy Diet Index (pHDI) (33.59 ± 10.32 in the first year vs. 30.07 ± 10.89 after a year, *p* = 0.02). The comparison between the bachelor’s students at different levels and the professionals (fourth year) revealed a significant difference in nutritional knowledge (20.08 ± 2.50 points in professionals vs. 16.31 ± 2.98 in the first-years and 16.86 ± 3.19 points in the second year of education, *p* < 0.001). There were no significant differences regarding nutritional behaviors and BMI between these groups. The post hoc analysis revealed a 100% power for the comparison of nutritional knowledge (first-years vs. professionals) and 99.8% for second-years vs. professionals. The sample size calculation (alpha = 0.05, power = 0.8) indicates that groups of n = 11 would be enough to support this analysis for the comparison between the first-years and the professionals, and n = 17 for the comparison between the second-years and the professionals. The detailed results are presented in Table 1. The analysis of the linear relationship revealed a moderate correlation between nutritional education level and nutritional knowledge (R = 0.48, *p* < 0.001). The detailed results are presented in Table 2.

### 2.3. Specific Product Intake

The analysis of specific healthy and unhealthy products’ intake revealed that professionals eat significantly more fast food, white meat, and less legumes and drink significantly less fruit and vegetable juices, soft and energy drinks, and alcohol compared to younger students. The detailed results are presented in Table 3.

The detailed analysis revealed a linear relationship between education levels and the mean intake of selected products; a positive weak correlation for white meat consumption (R = 0.28 *p* = 0.004); and a negative weak correlation for fruit juice (R = −0.23 *p* = 0.02, vegetable juice (R = −0.22 *p* = 0.02), and alcohol consumption (R = −0.21 *p* = 0.03). The detailed results are presented in Table 4.

### 2.4. Self-Assessment

The students and professionals were also asked to self-assess their nutritional knowledge level and their eating behaviors. The analysis revealed that the self-assessment results did not match the DQI (R: −0.03; 95% CI: −0.025 to 0.165; *p* = 0.80). On the other hand, the respondents correctly assessed their nutritional knowledge levels, as they matched the test’s results (R: 0.22; 95% CI: 0.032 to 0.394; *p* = 0.02).

### 2.5. Sleep Length

Most participants received a proper amount of sleep, as only 15.9% of the participants slept less than 6 h during weekdays, and only 5.7% of participants slept less than 6 h during weekends. It is notable that the participants tended to sleep longer during weekends compared to weekdays. The relationship between educational level and sleep length was not significant. On the other hand, there was a significant inverse correlation between the knowledge level and the length of sleep during weekends (R: −0.24, 95% CI: −0.428 to −0.032, *p* = 0.02). Moreover, the weekend’s length of sleep was also inversely correlated with nutritional indexes: the DQI (R: −0.32, 95% CI: −0.493 to −0.114, *p* = 0.003) and the pHDI (R = −0.28, 95% CI: −0.460 to −0.072, *p* = 0.01). The analysis of the relationship between the length of sleep and the consumption of selected drinks did not present any significant correlations for the consumption of tea and coffee, energy drinks, or alcohol. The analysis of the structure of sleep length during different years of education revealed a significant drop in the length of sleep during weekends between the first and second years of education. There were no significant differences observed later on.

### 2.6. Physical Activity

During their work and study time, 46.6% of the participants declared a low level of physical activity, 48.9% moderate, and 4.5% high levels of physical activity. However, in their free time, only 14.8% had low, 55.7% had moderate, and 29.5% had high physical activity levels. The analysis of the relationship between educational level and nutritional knowledge did not present any significant correlations. Moreover, BMI also was not correlated to physical activity levels, both at work and during free time. However, the analysis of the relationship between individual nutritional behaviors revealed a positive correlation between the pHDI (R: 0.34; 95% CI: 0.146 to 0.517; *p* = 0.001) and the DQI (R = 0.27; 95% CI: 0.59 to 0.500; *p* = 0.01) and physical activity levels during free time.

## 3. Discussion

The field of nutrition and dietetics has a very important place in the maintenance of health and the treatment of various diseases. Dietetics professionals are often responsible not only for nutritional counseling but also for overall lifestyle advice. As proper sleep hygiene and physical activity are also parts of healthy behavior, it is important that dietetics do not neglect these aspects. For this reason, the individual performance of students who receive education in this field is also important [27]. Another interesting aspect is that, sometimes, a student’s motivation to enter a specific field, e.g., dietetics, can be based on their own need for a personal change. That is why we wanted to estimate whether the academic knowledge gained throughout university education does in fact impact individual lifestyle behaviors.

The present study found differences in the dietary behaviors of dietetics students and professionals assessed with a validated tool. In this study, a linear correlation was found in the general nutritional knowledge of dietetic students and professionals, but it was not correlated with individual nutritional behaviors or BMI. Similarly to the result of our study, no association was observed between students’ knowledge on nutrition and their BMI in previous studies [20,21,28]. These findings are consistent with previous studies that show that health professionals, including medical students and physicians, may adhere to optimal dietary behaviors. In a study on the nutritional knowledge of medical students, the mean nutritional knowledge score of second-year students was found to be higher than that of first-year students [29]. In contrast to this result, previous studies have found inconsistent results on optimal dietary behaviors [30,31]. In a previous study, there was a significant negative, though moderate, correlation between years in practice and nutritional knowledge [17]. Additionally, the current study found that dietetics professionals reported higher knowledge scores compared to dietetics students. This is in line with previous research demonstrating that general higher education levels are associated with better nutritional knowledge [27,28]. In another study, nutrition students in higher semesters have significantly better nutritional knowledge compared to students in first semesters and students in other departments [32]. Moreover, a recent study focused on a similar topic in adolescents revealed a low level of nutritional knowledge and improper dietary behaviors among them [33]. However, over the course of this mentioned study, the authors did not analyze the potential relationship between these two aspects.

The differences in dietary behaviors and knowledge between dietetics students and professionals may reflect changes in attitudes and beliefs that occur during the transition from student to professional. This highlights the importance of ongoing education and professional development for dietetics professionals to ensure that they are equipped with the most up-to-date knowledge and skills to provide effective nutrition advice to clients.

The present study also identified specific food products that differed between the dietetics student and professional groups, including meat, beverages, fruits, and vegetables. In our study, the results of the analysis of specific healthy and unhealthy product intake revealed that professionals consumed more white meat and fast food, whereas their consumption of legumes, fruit and vegetable juice, soft drinks, energy drinks, and alcohol was lower than that of students. A possible reason for this is that entering professional work results in less time for food preparation. Another aspect is that, nowadays, fast food are not the most affordable option, so when the economic status of professionals improves compared to students, together with their lack of time, it can result in higher fast-food consumption. Similarly, the trends of white and red meat consumption could be associated with the economic change accompanying the transition from a student to a professional. Professionals can afford to eat more meat, which is considered an expensive product; however, combined with general nutritional consciousness, it could lead to an increase only in white meat consumption. According to the results of another study in which the food consumption frequencies of first- and fourth-year dietetics students were examined, it was observed that the frequency of consumption of milk, kefir, eggs, cheese, red meat, legumes, vegetables, fruits, whole wheat bread, bulgur, oatmeal, and oil seeds was higher in fourth-year students than in first-year students [27]. On the contrary, it was found that the frequency of consumption of foods such as sausages, salami, white bread, breakfast cereals, pastry, cookies, margarine, chocolate, sherbet desserts, hamburgers, pizza, and ready-to-drink beverages was higher in first-year students [27]. The differences in the results might be caused by different level of details acknowledged by the studies. The mentioned study was based on a non-validated questionnaire, which was not described or attached by the authors, while in our study, we used a validated tool. It is important to note that the presented study included prospective aspects of nutritional behavior monitoring throughout a year of education. In another study, in which the knowledge of dieticians and dietician candidates about nutrition was evaluated, it was found that students who received nutrition education consumed meat and dairy products more than students who did not receive nutrition education, while students who did not receive nutrition education preferred foods in the fat group more [34]. In a study on the nutritional knowledge of secondary-school adolescents, nutritional knowledge was independently associated with a lower intake of meat and iced tea and a higher intake of vegetables and plant-based oils. In addition, we observed that a higher knowledge level was associated with higher vegetable and oil consumption. Furthermore, our findings illustrate a positive association between nutrition education hours per week and healthier food choices, similarly to other studies, for example, a higher intake of whole grains and a lower intake of meat and energy drinks [35].

What is more, this study showed that sleep length was not related to educational levels overall; however, there was observed a significant drop in the length of sleep during the weekend between the first and second years of education. On the other hand, individual knowledge was inversely correlated with the length of sleep during weekends, as well as the nutritional dietary indexes, the pHDI and DQI. This suggests that the level of professional knowledge is not represented in the area of sleep hygiene. It is worth noting that the more individuals were educated and the healthier their individual diet was, the more they neglected the role of sleep in their own lives. This is important, as the length of sleep is associated with mortality and the incidence of civilizational diseases such as hypertension or obesity [12,13,14]. The analysis showed a significant drop in sleep length between the first and second year of education, without significant changes later. This observation is in line with the recent findings in first-year medical students, who also presented a drop in sleep quality at the beginning of their education [36]. This could be a result of the increased study load, which can be overwhelming at a young age. The analysis of the main nutritional factors suggested to be related to the length of sleep did not show any correlation between the length of sleep and the consumption of energy drinks, alcohol, tea, and coffee, even though there is evidence from meta-analyses about the deterioration of sleep quality related to caffeine and alcohol intake [37,38]. The results of our study are in line with the available literature, as it is reported that anthocyanin-rich foods, such as tart cherries, but also kiwi or warm milk consumption, has a positive impact on sleep quality [39,40,41,42]. Aspects such as sleep hygiene knowledge also improved sleep quality, which suggests that dietary knowledge also goes along with overall lifestyle knowledge, including sleep hygiene [5].

In terms of physical activity, only 14.8% of the participants had a low physical activity level. It is a positive remark, as 150 min of moderate or 75 min of intense physical activity weekly is advised for civilizational disease prevention [43,44]. The analysis showed differences between physical activity levels at work, even though the participants represented similar backgrounds. The differences might be explained by different ways of commuting chosen by the participants. Moreover, some of the dieticians are also personal trainers, which requires higher physical effort at work. These trends were mitigated by the free-time physical activity, the level of which was higher than the ones at work. They were also correlated with nutritional behaviors, but not with educational levels. It shows that even students understand not only the role of nutrition, but also physical activity as part of a healthy lifestyle, and implement it into their own lives.

The findings indicate that education and learning have a significant impact on the food choices of nutrition and dietetics students. Overall, these findings highlight the need for ongoing education and professional development for dietetics professionals to ensure that they are equipped with the most up-to-date knowledge and skills to provide effective nutrition advice to clients. In addition, the results of this study suggest that promoting the consumption of plant-based diets, including nuts, seeds, and plant-based milk alternatives, may be beneficial for improving the dietary behaviors of dietetics students and professionals alike. Therefore, the importance of nutrition education is emphasized, and the aim is to direct students’ food preferences towards healthier options.

## 4. Material and Methods

**Figure 1:**
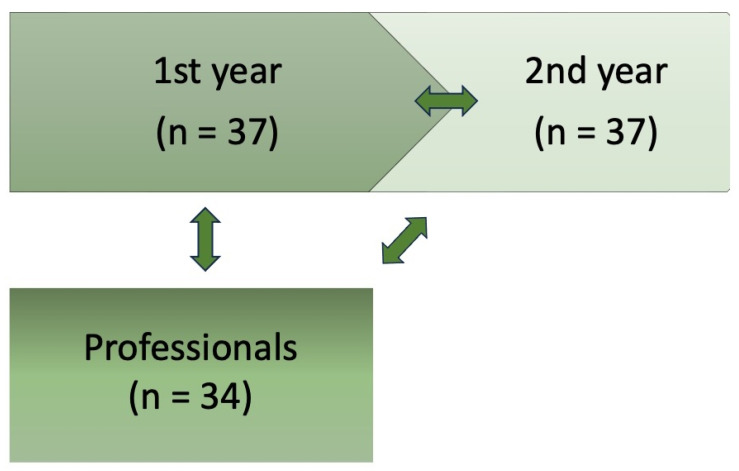
The flowchart of the analyses performed between groups.

A total of 71 dietetics students (37 bachelor’s and 34 master’s course students) were enrolled in this study. Recognized professional dietetics competencies in Poland are acquired at a university. A dietetics course is based on a bachelor’s degree, and then a bachelor’s graduate is already treated as a professional. Nonetheless, it can then be further voluntarily continued in a master’s degree course. That is why first-year bachelor’s degree students were included in a prospective year-long observation, while first-year master’s degree students (in the fourth year of education in dietetics) were treated as the professional group. The hypotheses were specified before the data were collected. A flowchart of the analyses performed between groups is presented in Figure 1.

General nutritional knowledge, sleep, and dietary behavior were assessed with a validated Questionnaire of Eating Behaviors [45]. It consists of four main parts. In the first part, general eating habits are assessed, while in the second part, the frequency of consumption of selected food products is analyzed. The respondents are asked about their typical consumption of selected products, and they can choose between options: never, 1–3 times/month, once a week, a few times a week, once daily, or a few times a day. Each response is then transferred into the mean daily consumption of each product, according to the guidelines of the data analysis within the validated Questionnaire of Eating Behaviors [45], as follows: never (0/day), 1–3 times/month (0.06/day), once a week (0.14/day), a few times a week (0.5/day), once a day (1/day), or a few times a day (2.0/day). On the basis of this, data quality indicators can be calculated. The Pro-Healthy Diet Index-10 (pHDI) is calculated by adding the mean daily consumption of 10 selected products recognized as healthy. The top score is 20. A result of 0–6.66 is interpreted as low, 6.67–13.33 as moderate, and 13.34–20.00 as high intensity. Analogically, the Non-Healthy Diet Index-14 (nHDI) is calculated by adding the mean daily consumption of 14 selected unhealthy products. The top score is 28. A result of 0–9.33 is interpreted as low, 9.34–18.66 as moderate, and 18.67–28.00 as high intensity. The pHDI and nHDI can be also expressed as a proportion (percentage) of the top score. This way, they can be compared to assess whether the dietary pattern presents the majority of healthy or unhealthy behaviors as the Diet Quality Index (DQI), which is the overall dietary pattern score calculated by subtracting the nHDI (range 0–100) from the pHDI (range 0–100). The score can be interpreted as an overall high intensity of unhealthy patterns (−100 to −26), a low intensity of overall unhealthy and healthy patterns (−26 to 26), and an overall high intensity of healthy patterns (26 to 100). The third part of the questionnaire reviews general views on food and nutritional knowledge. It is based on 25 statements regarding dietary knowledge. The respondent is asked to indicate whether each one is true or false, they can also choose the response “I don’t know”. For each good answer, the respondent is given one point, then the number of points (top score 25) is calculated. The results are interpreted as 17–25 for good dietary knowledge, 9–16 for fair dietary knowledge, and 0–8 for bad dietary knowledge. The expression of dietary behaviors and dietary knowledge in points allows for the comparison of individual nutritional behaviors among respondents and then for relating it to the level of general nutritional knowledge. The fourth part of the questionnaire deals with other lifestyle aspects, including sleep length during weekdays and weekends. The respondents were asked about how many hours of sleep they receive. The respondents could choose between options—less than 6 h, between 6 and 9 h, or more that 9 h—separately for weekdays and weekends. Moreover, the participants were asked about their physical activity level at work and during their free time. For their work time, they could choose between low (more than 70% of the time in a sitting position), moderate (about 50% of the time sitting and about 50% of the time moving), and high (approximately 70% of the time in motion or physically demanding work), while for their free time, they could choose between low (sitting, watching TV, reading newspapers and books, light housework, walking 1–2 h a week), moderate (walking, cycling, gymnastics, gardening, or other light physical activity performed 2–3 h a week), and heavy (cycling, running, gardening or other sports and recreational activities requiring physical effort performed more than 3 h a week).

The questionnaire was filled in by a group of 37 first-year bachelor’s degree students at the beginning of a dietetics course and after a year as a prospective observation. The questionnaire was also filled in by the group of 34 dietetics professionals participating in a master’s degree course (the fourth year of education in dietetics) as a comparative professional group. The dietary patterns of each participant were expressed with the DQI, pHDI, and nHDI, while dietary knowledge was assessed with the third part of the questionnaire as described above. The assessment of dietary patterns also included the analysis of the consumption of selected products in the questions. The level of education was expressed as the years of education in dietetics (one, two, or four).

This study was approved by the local Bioethics Committee of the Medical University of Lublin (consent no. KE-0254/9/01/2022). This study was conducted in line with the directives of the Declaration of Helsinki on Ethical Principles for Medical Research. All participants signed a written consent form.

Statistical analyses were performed with the RStudio software, v. 4.2.0. The normality of the distribution of each parameter was checked by the Shapiro–Wilk test. The variables were presented as means (±SD). Spearman rank correlation was used to investigate the relationship between the educational level, sleep length, and diet quality indexes or dietary knowledge test results. It was also used to analyze the relationship between the self-assessment and the objective results regarding nutritional knowledge and behaviors. The cut-off points used for the correlation coefficient were as follows: <0.40 as low, 0.40–0.69 as moderate, and ≥0.70 as high correlation. *p* values below 0.05 were considered significant [46]. Then, the differences in diet quality indexes or dietary knowledge test results between the groups of students at different levels and the professionals were investigated by the Mann–Whitney test. *p* values below 0.05 were considered significant.

## 5. Conclusions

This is the first study that prospectively analyzed sleep and nutritional behaviors and knowledge among dietetics students throughout their university education and compared it to already-trained professionals. The analysis showed that the length of sleep was inversely correlated with knowledge and nutritional indexes, which suggests that dietetics professionals tend to neglect this aspect of a healthy lifestyle. Moreover, the educational level was correlated with dietary knowledge; it was also properly self-assessed by the participants. However, the educational level and knowledge were not correlated with eating behaviors. The self-assessment of nutritional behaviors also did not correlate with the objective assessment. This study showed that a university education brings better nutritional knowledge to dietetics students and professionals; however, it does not impact their individual behavior regarding sleep and diet. It is important to underline that dietitians, apart from using their theoretical knowledge to guide their patients, should also implement their knowledge in their own everyday life.

### Strengths and Limitations of this Study

This is the first study that prospectively analyzed the complexity of lifestyle behaviors and knowledge among dietetics students throughout their university education and compared it to already-trained professionals. There are no other available studies that analyze all of these aspects together. It indicated that academic knowledge itself is not enough to change individual personal choices. On the other hand, despite the novelty of this study, it presents a limited number of participants, but the post hoc analysis showed that the number of participants was enough. However, the professionals and students were independent groups, so it would be very practical to conduct longer prospective analyses in this topic.

## Figures and Tables

**Table 1:** The comparison of nutritional knowledge, nutritional behavior, and BMI between different levels of education (DQI—Diet Quality Index, pHDI—Pro-Healthy Diet Index, nHDI—Non-Healthy Diet Index, BMI—body mass index).

	1st Year (n = 37)		2nd Year (n = 37)		1st vs. 2nd Year	Professionals (n = 34)		2nd-Years vs. Professionals	1st-Years vs. Professionals
	Mean	SD	Mean	SD	*p*	Mean	SD	*p*	*p*
Nutritional knowledge [points]	16.31	±2.98	16.86	±3.19	0.24	20.08	±2.50	<0.001	<0.001
DQI [points]	21.56	±12.17	19.18	±11.15	0.21	20.98	±13.15	0.38	0.98
pHDI [points]	33.59	±10.32	30.07	±10.89	0.02	32.45	±10.50	0.28	0.65
nHDI [points]	12.03	±5.90	10.89	±5.43	0.14	11.48	±5.43	0.64	0.79
BMI [kg/m^2^]	21.39	±4.95	21.87	±3.32	0.32	21.85	±2.31	0.93	0.97

**Table 2:** The correlation between education level and nutritional knowledge and behaviors (DQI—Diet Quality Index, pHDI—Pro-Healthy Diet Index, nHDI—Non-Healthy Diet Index, BMI—body mass index).

	R	*p*
Nutritional knowledge [points]	0.48	<0.001
DQI	−0.01	0.99
pHDI	−0.01	0.86
nHDI	−0.02	0.81
BMI	−0.02	0.85

**Table 3:** The comparison of selected products’ mean intake at different levels of education.

	1st Year (n = 37)		2nd Year (n = 37)		1st vs. 2nd Year	Professionals (n = 34)		2nd-Years vs. Professionals	1st-Years vs. Professionals
	Mean	SD	Mean	SD	*p*	Mean	SD	*p*	*p*
Fast food	0.10	±0.16	0.08	±0.08	0.36	0.11	±0.34	<0.001	<0.001
Processed meat	0.27	±0.29	0.23	±0.26	0.66	0.36	±0.43	0.11	0.28
Red meat	0.10	±0.15	0.09	±0.14	0.63	0.12	±0.14	0.11	0.28
White meat	0.30	±0.37	0.25	±0.28	0.66	0.51	±0.35	<0.001	0.001
Fish	0.13	±0.16	0.12	±0.19	0.57	0.16	±0.15	0.02	0.08
Legumes	0.38	±0.49	0.22	±0.25	0.07	0.20	±0.24	0.25	0.02
Fruit	1.37	±0.71	1.17	±0.67	0.27	1.12	±0.71	0.70	0.16
Vegetables	1.64	±0.60	1.65	±0.58	0.94	1.68	±0.61	0.78	0.74
Sweets	0.40	±0.39	0.38	±0.42	0.78	0.44	±0.42	0.26	0.54
Fruit juices	0.30	±0.32	0.27	±0.30	0.64	0.14	±0.25	0.03	0.02
Vegetable juices	0.15	±0.23	0.17	±0.27	0.71	0.05	±0.06	0.10	0.04
Sweetened hot drinks	0.70	±0.83	0.81	±0.82	0.35	0.80	±0.90	0.49	0.93
Soft drinks	0.18	±0.36	0.24	±0.49	0.94	0.06	±0.12	0.04	0.01
Energy drinks	0.18	±0.28	0.21	±0.27	0.35	0.08	±0.20	0.002	0.01
Water	1.88	±0.43	1.94	±0.23	0.62	1.82	±0.44	0.19	0.45
Alcohol	0.13	±0.16	0.10	±0.13	0.65	0.06	±0.09	<0.001	<0.001

**Table 4:** The correlation between education level and selected products’ consumption.

	R	*p*
Fast food	0.02	0.86
Processed meat	0.12	0.22
Red meat	0.07	0.47
White meat	0.28	0.004
Fish	0.08	0.41
Legumes	−0.19	0.05
Fruit	−0.13	0.18
Vegetables	0.03	0.79
Sweets	0.05	0.60
Fruit juices	−0.23	0.02
Vegetable juices	−0.22	0.02
Sweetened hot drinks	0.04	0.67
Soft drinks	−0.16	0.10
Energy drinks	−0.18	0.07
Water	−0.08	0.43
Alcohol	−0.21	0.03

## Data Availability

The data that support the findings of this study are available from the corresponding author upon reasonable request.
